# Glandular Trichomes and Essential Oils Variability in Species of the Genus *Phlomis* L.: A Review

**DOI:** 10.3390/plants13101338

**Published:** 2024-05-13

**Authors:** Irina Neta Gostin, Cristian Felix Blidar

**Affiliations:** 1 Faculty of Biology, “Alexandru Ioan Cuza” University of Iași, Bdul Carol I, No. 11, 700506 Iasi, Romania; 2 Department of Biology, Faculty of Informatics and Sciences, University of Oradea, Street Universităţii No. 1, 410087 Oradea, Romania; cblidar@gmail.com

**Keywords:** secondary metabolites, monoterpenes, sesquiterpenes, *Phlomis*, capitate trichomes, dendroid trichomes

## Abstract

The genus *Phlomis* is one of the largest genera in the Lamiaceae family and includes species used since ancient times in traditional medicine, as flavoring for food and as fragrance in cosmetics. The secretory structures (represented by glandular trichomes) as well as the essential oils produced by them constitute the subject of this review. While representatives of this genus are not typically regarded as large producers of essential oils compared to other species of the Lamiaceae family, the components identified in their essential oils and their biological properties necessitate more investigation of this genus. A comprehensive analysis of the specialized literature was conducted for each of the 93 currently accepted species to identify all the results obtained by researchers regarding the secretory structures and essential oils of this genus up to the present time. Glandular trichomes, still insufficiently studied, present morphological peculiarities that differentiate this genus within the family: they are of two categories: capitate (with a wide distribution in this genus) and dendroid. The peltate trichomes, characteristic of many species of this family, are absent. The essential oils from the species of the genus *Phlomis* have been much more widely studied than the secretory structures. They show considerable variability depending on the species and the environmental conditions.

## 1. Introduction

Glandular trichomes, which are found in almost one-third of plant species [1], play an important role in the life of plants by serving as specialized structures for the production and storage of various substances, including essential oils and other secondary metabolites [2]. These substances can act as a defense mechanism against biotic and abiotic stresses [3], thereby helping plants to survive and thrive in their natural habitats. Additionally, glandular trichomes can also attract pollinators [4] and deter pests [5], contributing to the reproductive success and overall fitness of the plant species.

The genus *Phlomis* (family Lamiaceae) includes 93 species (excluding hybrids and subspecies) accepted today and spread over three continents, Asia, Europe and Africa, in temperate or subtropical climates. This is according to the current data on Plants of the World Online (POWO) [6] (https://powo.science.kew.org; accessed on 18 February 2024), administered by the Royal Botanic Gardens, Kew, UK and World Flora Online (WFO) [7] (https://about.worldfloraonline.org; accessed at 18 February 2024), These databases were considered in carrying out the investigations in this paper.

From a taxonomic point of view, the genus *Phlomis* belongs to the family Lamiaceae (Lamiales) [8], the subfamily Lamioideae and the tribe Phlomideae (which includes the genera *Phlomis* and *Phlomoides*) [6,9]. The genus is monophyletic [10], including perennial species [11]. The phylogenetic studies carried out in the last two decades led to the inclusion of a significant number of species from the genus *Phlomis* (which now contains only sub-shrubs or shrubs) in the genus *Phlomoides* (with herbaceous species) (today, they are accepted as distinct genera [12], after numerous controversies that followed their separation by Moench in 1794) [11]. Azizian and Cutler [13] found anatomical and morphological affinities between the species belonging to the genera *Phlomis* and *Eremostachys*. But later, most of the species of this genus were included in the genus *Phlomoides* [11]. Relevant is the example of the species *Phlomoides tuberosa* Moench, removed by Moench in 1794 [11] from the genus *Phlomis*, reintroduced by Bunge in 1830 [14] in the genus *Phlomis* (*P. tuberosa* L.) and resettled by Mathisen et al. [11] in the genus *Phlomoides*.

The species of plants from the Lamiaceae family have been used since ancient times by people in medicine, food, hygiene, cosmetics and agriculture due to their secondary metabolites and, primarily, due to their essential oils [15,16]. They are found in large quantities and with varied compositions in most of the species belonging to this family [2]. The essential oils from the *Phlomis* species have antibacterial activity: for example, the oil of *Phlomis lanata* [17] and *P. salicifolia* [18] acts especially on the Gram-negative bacteria *Escherichia coli* and *Pseudomonas aeruginosa*, the oil of *P. fruticosa* [19] and *P. olivieri* [20] showed an antibacterial effect against both Gram-positive and Gram-negative bacteria (*Bacillus subtilis* and *E. coli*) and the oil of *P. rigida* was active against the Gram-positive bacteria *Staphylococcus aureus* [21]. The antifungal effects of oils from various *Phlomis* species have also been investigated; those from *P. cretica*, *P. samia* [22], *P. lanata* [17] and *P. rigida* [21] have been proven to exert antifungal action on some pathogenic species of *Candida* sp.

Essential oils extracted from certain species of the *Phlomis* genus have demonstrated potent antioxidant properties. For instance, the oil derived from *P. bourgaei* [23] and *P. pungens* var. *pungens* [24] displayed notable metal chelation activity, while the oil of *P. armeniaca* exhibited a significant reducing capacity in the presence of ferric and cupric ions [24]. The effect of the inhibition of α-amylase enzyme activity by *P. nissolii* essential oil and of α-glucosidase by *P. armeniaca* oil [24] can be associated with the use of these species in the treatment of diabetes [25].

Glandular trichomes are considered true “natural biofactories” [2] essential oils but are also for other secondary metabolism compounds, their secretion being polymorphic, depending on the species and their structure [26]. Although there are numerous studies on the morphology and histochemistry of glandular trichomes in species of the Lamiaceae family [27,28,29,30,31,32], the number of research works that refer to the *Phlomis* genus remains quite limited.

According to the data available in the literature, trichomes from the species of the Lamiaceae family are of two major types: peltate and capitate [1,33,34,35]. Peltate trichomes are formed by a basal cell, a foot cell and a variable number of secretory cells, arranged in a single plane [36]. They are usually specialized in the secretion of essential oils, which they store in the subcuticular space. Capitate trichomes have a basal cell, one or more stalk cells (of variable lengths) and one to four (rarely more) glandular cells [37]. Often, their secretion is mixed, with a variable structure. Sometimes, it can consist exclusively of hydrophilic compounds (as, for example, in some capitate trichomes from *Salvia officinalis* [28]), but in some cases (especially in species that do not present peltate trichomes), they can mainly secrete essential oils [38]. Besides the biological role of producing essential oils, glandular trichomes [39,40] (along with non-glandular ones) [41] have an important role in the taxonomic delimitation of species and genera from the Lamiaceae family.

The purpose of this review is to provide up-to-date information on the state of research on glandular trichomes and the composition of essential oils from all currently recognized *Phlomis* species. The research concerned querying the Web of Science, Scopus and Google Academic databases with keywords consisting of the scientific names of the 93 *Phlomis* species accepted (as well as their synonyms, used in the past), plus “glandular trichomes”, “glandular hairs”, “secretory hairs” and “essential oil”, in order to identify papers that contain information about these aspects. In the case of essential oils, since the chemical composition of the product extracted from the leaves or aerial parts at anthesis is analyzed in most cases, this was considered in the present study; for each case, the first five compounds of the essential oil, in descending order of concentration, were mentioned in the synthetic table.

## 2. Results

### 2.1. Glandular Trichomes in the Phlomis Genus

The secretory trichomes of species from the Lamiaceae family have been investigated from morphological, histochemical and ultrastructural points of view [27,28,29,30] in an attempt to understand as precisely as possible the mechanisms of synthesis of the secondary metabolites elaborated by them. But for the *Phlomis* genus, compared to the total number of species currently accepted, the number of species for which there are data (complete or partial) in the literature remains small (13 species out of 93 in total).

#### 2.1.1. Structure of the Glandular Trichomes

**Figure 1:**
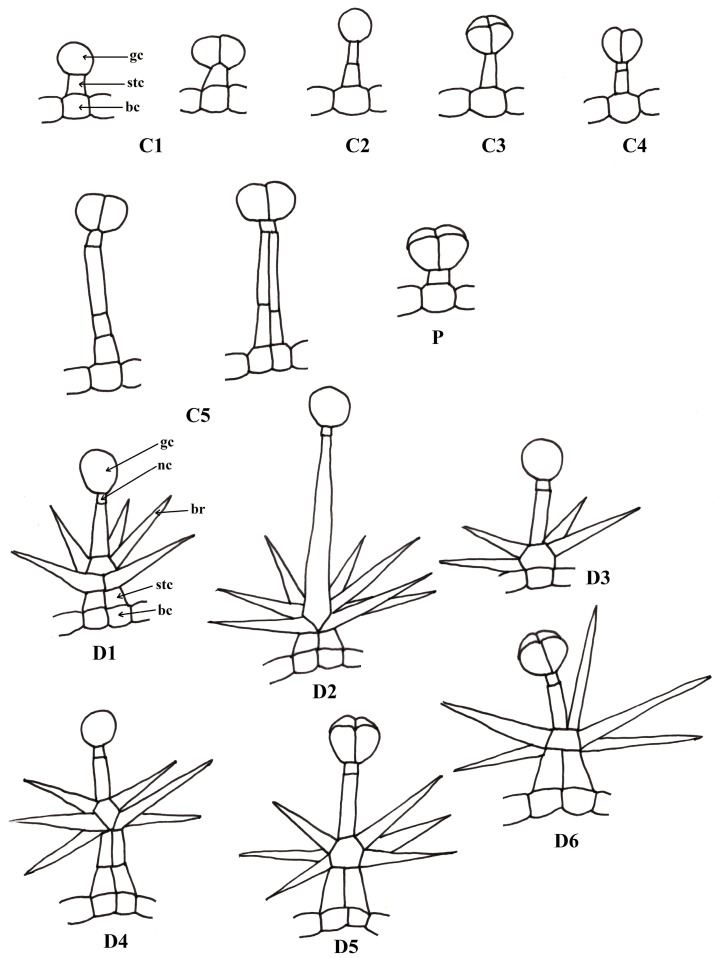
Types of glandular trichomes described in some species of the genus *Phlomis*: bc—base cell, stc—stalk cell, gc—glandular cell, br—branch, nc—neck cell.

The data available in the literature regarding the morphology of glandular trichomes in the *Phlomis* species are synthesized in Table 1 and Figure 1. Considering the morphological characters described by various authors [13,39,42,43,44,45,46,47,48,49], we grouped the capitate trichomes into five categories (C1–C5) and the dendroid trichomes also into six categories (D1–D6). There is still variability in relation to these categories; the classification was made to simplify the description. In establishing the subtypes, the number of secretory cells, the relative size of the stalk, the number of component cells and the positioning and density of the branches (in the case of dendroid trichomes) were taken into consideration.

The following types have been described: capitate glandular trichomes—C1: 1 basal cell + 1 stalk cell + 1–2 glandular cells; C2: 1 basal cell + 2–3 stalk cells + 1 glandular cell; C3: 1 basal cell + 1 stalk cells + 4 glandular cells; C4: basal cell + 1–2 stalk cells + 2 glandular cells; C5: uni- or biseriate stalk with 4–5 cells + 2 glandular cells; dendroid glandular trichomes—D1: 4–10 branches; D2: 7–9 branches, long stalk cell; D3: branches inserted at the base + 1–2 glandular cells; D4: branches inserted in the median area + 1–2 glandular cells; D5: branches inserted in the median area + 4 glanduar cells; D6: mixed + 4 glandular cells and non-glandular branching at the top; P: glandular trichome described as a peltate, reclassified in the capitate trichome category with 4 glandular cells (C3).

Azizian and Cutler [13] describe four forms of capitate trichomes, which correspond to C1 (form 1, found in many of the studied species: *P. aurea*, *P. chimerae*, *P. lanata*, *P. samia*), and C4 (form 2: *P. chimerae*) and C5 (form 3, 4: *P. crinita*). Type C2 is described by El-Banhawy and Al-Juhani [42] in *P. aurea* (distinct from type C1, consisting of short trichomes, but also from C3 or C4, because it has a single large glandular cell). Type C3 is described in *P. aurea* [42], *P. herva-venti* [46] and *P. fruticosa* [39] and has four large glandular cells.

Dendroid glandular trichomes are very rarely described in the literature, but they are not found only in Lamiaceae species; Gangaram et al. [50] describe a similar type of trichome in *Barleria albostellata* C.B. Clarke and Ahmad [51] describe them in *Dyschoriste vagans* (Wight) Kuntze, from the Acanthaceae family, a family related to the Lamiaceae, both belonging to the Lamiales order [52]. The term “dendroid glandular trichome” was used by Nikolakaki and Christodoulakis [44], Yetişen [48], El-Banhawy, Al-Juhani [42] and Gostin [46]. These trichomes were also described as “compound glandular hairs” by Azizian and Cutler [13], “stellate type with glandular arms” by Çalı [49] and “branched stalked” by Giuliani [39]. We chose to use this terminology due to the fact that the non-secretory part of the trichome is clearly dendroid, an accepted term for non-glandular trichomes in the same category [53].

El-Banhawy and Al-Juhani [42] describe two subtypes of dendroid trichomes, which correspond to D1 and D2 (in *P. aurea*). Azizian and Cutler [13] describe a type of dendroid trichome branched directly from the base (D3) in *P. brevilabris*, also found sporadically in *P. herba-venti* [46]. D4 is the most frequent type of dendroid trichome (long-legged, multiseriate) (*P. crinita*, *P. fruticosa* and *P. monocephala*) [43,44,48]. The last two types, D5 and D6, which present four glandular cells, were described in *P. oliveri* and *P herba-venti* [46,48].

Peltate glandular trichomes were reported in two species of the genus *Phlomis*–*P. oliveri* and *P russeliana*. In the literature, peltate trichomes are defined as having a basal cell, a short leg and a secretory head consisting of 4–12 cells that are covered by a common cuticle [54]. In the absence of clear characters that differentiate the peltate trichomes from the capitate ones from a morphological point of view, their classification in one category or another by some authors remains controversial. Werker [33] leaves the possibility of being included in the category of capitate trichomes and those with four secretory cells, of different sizes; peltate trichomes (also called “glandular scales”) should have secretory cells flattened in a horizontal plane. Muravnik [35] describes the petaloid trichomes, characteristic of the Lamiaceae species, as having “a disk-shaped head”. The research of Azizian and Cutler [13] on the trichomes of *Phlomis lanata* and *P. chimerae* place trichomes with four secretory cells in the “capitate” category, not the “peltate” category. Therefore, they investigate the species *P. russeliana* and do not mention the “peltate trichomes” category related to it.

Referring to these observations, and taking into account that in the case of the other species of *Phlomis* investigated regarding the presence of secretory trichomes, the “peltate trichomes” category is not described, we consider that future investigations (micromorphological, anatomical and histochemical) are necessary to clarify the presence or absence of peltate trichomes in *P. oliveri* and *P. russeliana*.

One of the problems encountered in the inventory of the categories of glandular trichomes was the illustrations in some analyzed papers (which were insufficient or of poor quality); this did not always allow an objective evaluation of the type of trichome described by the authors, it being necessary to take into account the authors’ description. Without sufficient photo documentation, these data must be considered cautiously, as a reevaluation of the species is necessary from this point of view.

#### 2.1.2. Secretion of Glandular Trichomes

Although they are the most widespread within the Lamiaceae family and responsible for the production of significant amounts of essential oils, peltate trichomes are absent in species of the *Phlomis* genus. Their role in the secretion of volatile oils has long been considered major within the family, some authors [33] considering species lacking peltate trichomes as not belonging to the category of aromatic plant species (for example, *Prasium majus* L.). However, the progress of research on the histochemistry of glandular trichomes indicated the presence of secretion consisting of essential oils in capitate and dendroid trichomes [39,46].

The genus *Phlomis*, along with other genera like *Sideritis*, *Lycopus*, *Micromeria* [55], *Marubium* and *Balota* [40], are not known for being the most abundant producers of essential oils within the Lamiaceae family. However, despite their relatively lower quantity of essential oils compared to some other Lamiaceae species, the essential oils derived from these genera are recognized for their valuable bioactive compounds. *Phlomis* species also present other secondary metabolites (besides the essential oil components) for which these species are utilized for medicinal purposes, including iridoid glucosides, flavonoids, phenylethanoid glycoside [25,56], phenylpropanoids and phenolic acids [57]. These compounds have been utilized in traditional medicine practices for centuries, indicating their historical significance and therapeutic potential [58,59]. Despite their long history of use, the full range of their medicinal properties and applications has yet to be fully explored and exploited in modern medicine and pharmacology.

The classification made by Werker [33], depending on the timing of secretion, which groups the secretory trichomes into short-term glandular hairs (capitate) and long-term glandular hairs (peltate), cannot be applied to the species of the genus *Phlomis*. Being devoid of peltate trichomes, the synthesis of essential oils is localized at the level of capitate and dendroid trichomes, which show continuous activity also on mature leaves [38,44,48] (where they are found also in the secretory phase and not only the post-secretory one).

Unlike the peltate trichomes, the capitate ones present much more varied secretion products. In *Phlomis herba-venti*, in C2-type capitate trichomes, visible drops’ essential oils were identified (by staining with the NADI reagent and Sudan III) [46], while C1-type trichomes have a mixed secretion containing, besides lipids, phenolic compounds and polysaccharides (identified by staining with toluidine blue and Ruthenium Red with the PAS reagent) [46]. The capitate trichomes of type C1 from *P. fruticosa* show only hydrophilic secretion (polysaccharides and mucopolysaccharides), being positive when stained with Ruthenium red and Alcian blue [39]. At the same time, C4-type trichomes accumulate terpenes, polyphenols and flavonoids, showing strong positive reactions to Fluoral Yellow-088, NADI reagent and aluminum trichloride. A similar reaction was shown by the dendroid trichomes (D4 type) described in this species [39].

Positive reactions for compound terpenes and phenolics were also observed in type C1 and C4 trichomes from the *Phlomis fruticosa* species [44]. The essential oil accumulates as droplets in the space between the cuticle and the external wall of the glandular cells [44]; this space stores phytotoxic compounds, serving as a primary defense mechanism at the plant’s surface [60]. The subcuticular space was observed primarily in capitate trichomes, while the extrusion of secretion products in dendroid trichomes typically occurs through the cell wall and the cuticle, into the external environment [46].

Dendroid trichomes also present mixed secretions, with lower amounts of essential oils than capitate ones; in *Phlomis herba-venti*, the glandular cells of these trichomes showed positive reactions to phenolic compounds, sesquiterpenes, polysaccharides and lipids [46].

Neck cells were observed in all dendroid glandular trichomes, often recording the same positive histochemical reactions as with secretory cells or secretory products [44]. They represent a special structure, with properties different from those of ordinary stalk cells, compared to which they are considerably shorter. The neck cells are involved in the secretion process, and there is communication with the neighboring cells, as shown by the ultrastructural studies performed on the capitate trichomes from *Stachys heraclea* All. [61]. Their lower transverse walls are cutinized [35], the structure being similar to Casparyan strips from the root or stem. In this way, the flow of substances is controlled (especially secondary metabolites secreted by the cell/glandular cell + neck cell complex), preventing the reflux towards the rest of the stalk cells [46] and the dissipation of the active substances in the trichome body. This structural peculiarity was also observed in secretory trichomes from other species of Lamiaceae [62].

The branch cells of dendroid trichomes are alive at their full development; in confocal microscopy observations, viable chloroplasts were observed in all these cells in *P. herba-venti* [46]. Non-glandular trichomes from Lamiaceae do not represent inert structures, with only a protective role against physical factors, but produce various categories of substances (proteins, lipids, terpenes, alkaloids, phenolic compounds and polysaccharides) that modulate the interactions between plants and other species from ecosystem. They complement the active protective role that glandular trichomes have for the plant [41].

The main role of glandular trichomes is to protect plants against herbivores: as a result, species that present trichomes (both glandular and protective) are less consumed by herbivores or attacked by parasites, a fact observed by researchers in individuals of the same species that present polymorphism for trichome production [63]. Moreover, *Phlomis* species are known to be little consumed by herbivores or attacked by phytophagous insects [64].

Second, trichomes located in the reproductive sphere (on sepals, petals or even ovaries) can play a role in attracting pollinators through the volatile substances they eliminate [65]; among the components found in the essential oil of *Phlomis* species, 1,8-cineole, linalool and (E, E)-α-farnesene have been proven to be attractive to various species of pollinating insects (Hymenoptera) [65] and bicyclogermacrene for some Diptera species [66].

### 2.2. Essential Oils from Phlomis Species

Species belonging to the *Phlomis* genus generally produce lower amounts of essential oils than other species from the Lamiaceae family [9]. However, the diversity of the component elements with valuable therapeutic properties or with the potential to be used in agriculture and industry makes a more careful evaluation of them necessary. The biological activity was observed both at the level of the essential oils as a whole and in the case of their components investigated separately [16]; many times, their effect was manifested synergistically [5].

Table 2 presents the existing data in the specialized literature regarding the composition of essential oils from all 93 recognized species. Because the geographical origin of the analyzed species is important, the area from which they are native and the predominant biome were indicated for each species (cf. POWO) [6]. Among the 93 species, complete or partial information on the composition of the essential oils was found for 48 (51.61%). The identification of the components of the essential oils was achieved by gas chromatography coupled to mass spectrometry (GC–MS) techniques. The lack of information for the other species leaves open a significant area of research in this field to cover the “white spots”.

The increased variability of the composition of essential oils is well known not only in the representatives of the Lamiaceae family [16] but also in those of other botanical families such as Asteraceae [122] and Lauraceae [123]. This fact is due both to genotypic variations (between individuals of the same species, which belong to different populations) as well as to environmental conditions or agrotechnical factors in the case of cultivated species [124,125]. Also, in the *Phlomis* genus, there is a high, well-known variability between the composition of the essential oils produced by the glandular trichomes on the leaves compared to those in the floral sphere [68,77], as well as between the oils produced in different stages of the ontogenetic development of plants [105].

The presence of different compounds in essential oils is part of a wider register of the modulation of interrelationships in ecosystems, between plants (immobile organisms) and various animal species (especially insects); mobile individuals have the role of performing various “services” for those in the first category, or, on the contrary, immobile plants must defend themselves against them, using biochemical signals because physical movement is impossible.

Analyzing the main compounds of the essential oils from 25 species of the genus *Phlomis* (of which 4 were reclassified in the genus *Phlomoides*: *P. younghunsbandii*, *P. szechuanensis*, *P. megalantha* and *P. umbrosa*), Amor [56] classifies them into four chemotypes: 1—which contains predominantly sesquiterpenes, 2—which contains both monoterpenes and sesquiterpenes, 3—which contains fatty acids, aliphatic compounds and alcohol and 4—which contains terpenes, fatty acids, aliphatic compounds and alcohol. Chemotype 3 includes only three species of those currently found in the genus *Phlomoides* (*P. younghunsbandii*, *P. szechuanensis* and *P. umbrosa*) [6].

#### 2.2.1. Sesquiterpenes from Essential Oils

**Figure 2:**
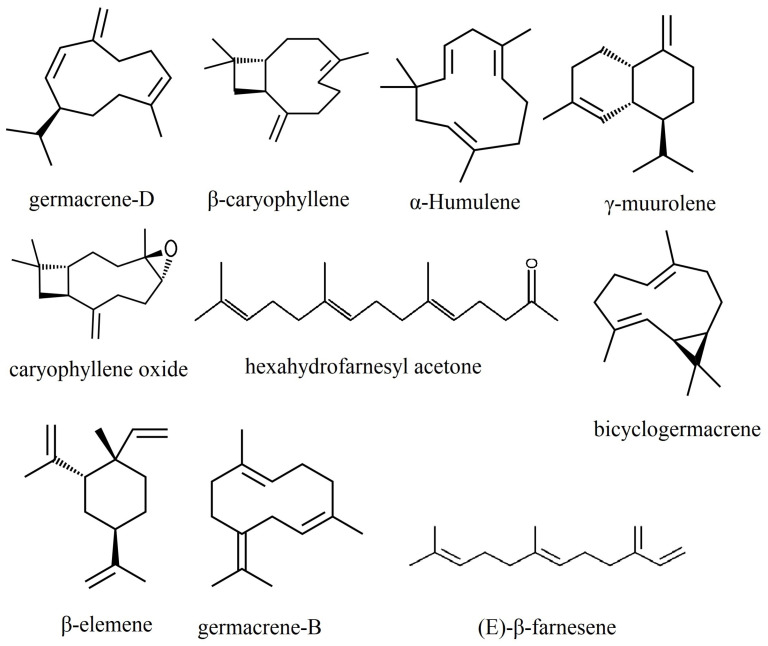
The main sesquiterpenes in the composition of essential oils of species of the *Phlomis* genus.

Among the components of the essential oils extracted from the leaves of species belonging to the genus *Phlomis*, sesquiterpenes comprise the largest share (Figure 2). Germacrene-D is one of the main components found in 31 out of the 47 species for which data are available in the literature, while β-caryophyllene is present in 25 species.

Germacrene-D was identified in the highest quantities in the species *Phlomis anisodonta* (65.0%) [58], *P. bruguieri* (60.05%) [78], *P. kurdica* (55.4%) [97] and *P. aurea* (51.56%) [74]. This compound was not among the first five components of essential oils in the species *P. brevibracteata* [77], *P. bucharica* [81], *P. cashmeriana* [85], *P. elliptica* [89], *P. lanata* [17], *P. lurestanica* [104], P. *platystegia* [76], *P. regelii* [115], *P. salicifolia* [81] and *P. thapsoides* [120]. Most of these species have, as their main component, a monoterpene or a fatty acid, which confirms the existence of some chemotypes within the species of this genus, as was pointed out by Amor [56].

β-caryophyllene and its oxidized form, caryophyllene oxide, are present among the first five components of essential oils from the majority of *Phlomis* species, with the exception of *P. aurea* [74], *P. brachyodon* [76], *P. bucharica* [81], *P. cashmeriana* [85], *P. lurestanica* [104], *P. monocephala* [67], *P. platystegia* [76], *P. salicifolia* [81] and *P. thapsoides* [120]. We notice that in six species of *Phlomis*, both germacrene-D and β-caryophyllene are missing from the main components of the essential oils. In these, the chemotypes are mainly based on monoterpenes and fatty acids.

Large amounts of β-caryophyllene are found in the species *Phlomis aucheri* (27.0%) [64], *P. bourgaei* (37.37%) [23], *P. chimerae* (31.6%) [86], *P. cypria* (37.4%) [77] and *P. rigida* (31.2%) [116], and large amounts of caryophyllene oxide are found in *P. aucheri* (33.5%) [64].

Some of the main constituents identified in the essential oil from various species of *Phlomis*—germacrene D and β-caryophyllene—are substances with a well-known deterrent role, which protects plants against herbivores [126]. β-farnesene is the main constituent of the essential oil of *Phlomis elliptica* (28.9%) [89] and *P. samia* (20.7%) [22], being part of the first 5 constituents of 15 other species of *Phlomis*. (E)-β-farnesene has an interesting biological role, being an alarm pheromone for insects from the Aphididae family [127]. It is emitted by aphids when they are attacked by enemies to warn individuals from the same group [128]. For this reason, (E)-β-farnesene acts as a repellent against these harmful insects, which avoid plants whose oil contains this compound. However, the repellent effect does not manifest equally against all insect species: Mumm and Hilker [129] showed that (E)-β-farnesene has an attractive effect against the wasp *Chrysonotomyia ruforum* Krausse (Hymenoptera, Eulophidae), an oophagous parasitoid for *Diprion pini* L. (Hymenoptera, Diprionidae).

#### 2.2.2. Monoterpenes from Essential Oils

**Figure 3:**
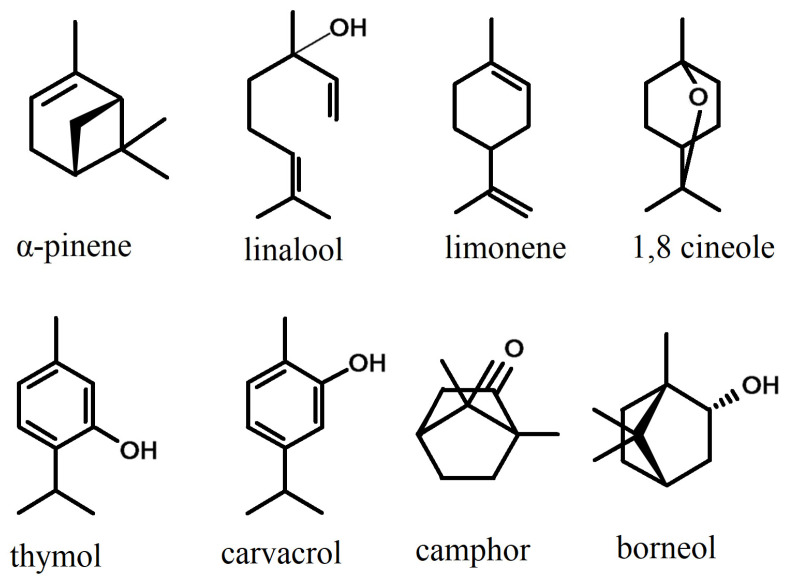
The main monoterpenes in the composition of essential oils of the species of the *Phlomis* genus.

Monoterpenes (Figure 3) are found less often and in smaller quantities in essential oils from the *Phlomis* species; however, the oils from some species proved to be richer in monoterpenes than in sesquiterpenes. Monoterpenes and their derivatives give flavor and aroma to the essential oils in which they are found [130].

Linalool is part of the group of acyclic monoterpenoids and represents an important component in essential oils for its pharmacological effects. Research has highlighted its antidepressant [131], immunomodulator and antimicrobial roles. It was indicated as the main component in the essential oil of *Phlomis leucophracta* (36.4%) [98], being also found in important quantities in the oils of *P. fruticosa* (8.0%) [22], *P. nissolii* (11.3%) [24], *P. cretica* (7.5%) [22] and *P. platystegia* (7.72%) [76].

Limonene is the main component of *Phlomis leucophracta* oil (14.56–27.86%), refs [70,99] observed this in two other populations distinct from the one investigated by Sarikurkcu et al. [98]. The essential oil from *P. leucophracta* possesses very strong antioxidant activity, similar to that of ascorbic acid, which denotes the increased and still unexploited potential of these species for use in the pharmaceutical and food industries [98,132].

Other monoterpenes found in large quantities in the essential oils extracted from the leaves are 1–8 cineole (15.9%) in *Phlomis regelii* [115], camphor (14.46%) in *P. bucharica* [81] and thymol in *P. bucharica* (20.41%) and *P. sewerzowii* (35.76%) [81]. In the last species, thymol together with carvacrol (8.9%) represent almost half of the components identified in the essential oil. Among the monoterpenes found in *Phlomis* species oils, α-limonene, pinene, camphor, linalool and borneol represent the compounds with the most significant aromatic properties [125].

1–8 cineole (also known as eucalyptol) is frequently found in the oil from different species of Lamiaceae: populations of *Lavandula angustifolia* from Brazil and *L. x intermedia* from Iran or Mexico make up between 31.6% and 47.94% of the composition of the essential oil from this compound [133]. In the *Phlomis* species, it is found in larger quantities in *P. bucharica* (13.69%) [81] and in *P. regelii* (15.9%) [115], both species being part of the predominant chemotype with monoterpenes. 1–8 cineoles have a strong anti-inflammatory and antioxidant effect [134], as well as an insecticide effect [135].

Thymol and its isomer, carvacrol [136], have phytotoxic, cytotoxic and genotoxic properties, being able to be used as selective bioherbicides [137]; they also have important antibacterial effects [138], being recommended even in the case of bacteria resistant to classic antibiotics.

Recent studies are increasingly highlighting the anticancer action of some monoterpenes; among those found in the composition of the oil of the *Phlomis* species, linalool shows cytotoxic, apoptotic and antiproliferative properties on breast cancer cells [139], α-pinene induces apoptosis in vitro on the human gastric adenocarcinoma cell-line (AGS) [140] and limonene acts on receptors involved in the chemoresistance of cancer cells [141].

#### 2.2.3. Other Compounds from Essential Oils

Hexadecanoic acid (palmitic acid) is the main component of *Phlomis* essential oils, apart from monoterpenes and sesquiterpenes. This is the dominant component from *P. herba-venti* (68.1%) [24], *P. cancellata* (17.13%) [84] and *P. elliptica* (19.1%) [64]. It is also found, among the main components, in *P. armeniaca* (4.9%) [67], *P. kurdica* (8.4%) [97], *P. lunariifolia* (9.7%) [103] and *P. tenorei* (12.8%) [119]. Hexadecanoic acid has an antibacterial and antifungal effect and can be used therapeutically in patients with asthma [142].

Methyl palmitate (the main component of *Phlomis salicifolia* oil) [81] has an effect similar to that of brood pheromone in honeybees [143]; these hormones are produced by larvae and trigger feeding instincts in nurse insects, including by increasing the amount of pollen collected from various species. This species, endemic to Central Asia, grows in a semi-arid habitat [81], where the pollinating insects are few in number, and the plant species have to make considerable “efforts” to attract them. Hexadecane (8.97%) is the main component of the essential oil from *Phlomis lurestanica*, an endemic species from the mountainous areas of Iran [144].

The presence of different compounds in essential oils is part of a wider register of the modulation of interrelationships in ecosystems, between plants (immobile organisms) and various animal species (especially insects); mobile individuals have the role of performing various “services” for those in the first category, or, on the contrary, immobile plants must defend themselves against them, using biochemical signals because physical movement is impossible.

## 3. Conclusions

The review of specialized literature aimed at identifying the results of the research conducted so far on the secretory structures and volatile oils from species of the *Phlomis* genus, which has highlighted the fact that the level of knowledge is still insufficient. If, in terms of the chemical composition of essential oils, 51.61% of the taxonomically accepted species have had their component elements described (even partially), the knowledge regarding glandular trichomes is limited to only 13 species (13.97%).

Although there is a substantial amount of information available regarding the essential oils from species of the genus *Phlomis*, future studies are needed to fully understand their composition. There are still 45 species whose essential oils remain completely unknown, and they may represent a potential source of biologically active compounds.

The genus *Phlomis* is unique among the genera of the Lamiaceae family because of the presence of a rare type of glandular trichomes, namely, dendroid glandular trichomes. They have from one to four secretory cells arranged on a stalk of a trichome morphologically similar to the non-glandular ones, with which it coexists in the indumentum on the vegetative or reproductive organs. But the current data on their morphology and structure are still very limited. Based on the data available so far, peltate trichomes are absent in species of the genus *Phlomis*. Investigations regarding the histochemistry of glandular trichomes (carried out in order to locate secretion products) are rare, and those regarding their ultrastructure are completely missing. Considering that trichomes, both glandular and non-glandular, serve as taxonomically significant traits for plants, it is imperative to conduct further investigations into species within the *Phlomis* genus in order to help clarify some classification and phylogenetic problems that exist in this taxon.

## Figures and Tables

**Table 1:** Types of glandular trichomes described in some species of the genus *Phlomis*.

Plant Species	Capitate Trichomes	Dendroid Trichomes	Author
*Phlomis aurea* Decne.	Type C1	Type D1	[42]
	Type C2	Type D2	
	Type C3		
*Phlomis brevilabris* Ehrenb. ex Boiss.	Type C1	Type D3	[13]
*Phlomis chimerae* Boissieu	C1		
	Type C4		[13]
*Phlomis crinita* Cav.	Type C1	Type D2, D4	[43]
	Type C5		[13]
*Phlomis fruticosa* L.	Type C1	Type D4	[44]
	Type C4		
	Type C1	Type D4	[39]
	Type C 3		
*Phlomis herba-venti* L.	Type C1	Type D3	[46]
	Type C3	Type D4	
		Type D5	
		Type D6	
*Phlomis kurdica* Rech.f.	Type C1		[45]
*Phlomis lanata* Willd.	Type C1		[13]
	Type C2		
	Type C3		
*Phlomis monocephala* P.H.Davis	Type C1	Type D3	[48]
	Type C2	Type D4	
	Type C5	Type D6	
*Phlomis olivieri* Benth.	Type C3 (P?)		[47]
	Type C4		
	Type C5		
		Type D3	[13]
*Phlomis rigida* Labill.		Type D2	[45]
*Phlomis russeliana* (Sims) Lag. ex Benth.	Type C3 (P?)	Type D1	[49]
	Type C2		
	Type C4		
*Phlomis samia* L.	Type C1		[13]

**Table 2:** The chemical composition of essential oils from species of the *Phlomis* genus (a synthesis). The first five components of the essential oil extracted from the leaves, in descending order of concentration (where available), were considered. (A dash ‘—’ in the table means that there is no information available about these species; for *Phlomis fruticosa* L., collected fromBar, Montenegro: locality A is exposed to the sun and locality B is in the forest).

Plant Species	Species Range/Biome (cf. POWO)	Biologically Active Chemical Compounds from the Essential Oil	Percentage Content	Collection Site	Author
*Phlomis amanica* Vierh.	Turkey (Hatay)/ subtropical	germacrene-D	14.17	Hatay, Haymaseki village, Turkey	[67]
		bicyclogermacrene	10.70		
		15-isopimaradien-11-ol	22.8		
		(Z)-farnesene	8.30		
		spathulenol	6.30		
*Phlomis angustissima* Hub.-Mor	SW Turkey/temperate	No data available.	—	—	—
*Phlomis anisodonta* Boiss	N. Iraq to Iran/ temperate	germacrene-D	65.0	Mazandaran province, Iran	[58]
		β-caryophyllene	11.0		
		bicyclogermacrene	2.30		
		β-elemene	2.60		
		trimethyl-2-pentadecanone	2.80		
		germacrene-D	52.60	Lorestan province, West Iran	[68]
		β-caryophyllene	15.90		
		α-pinene	6.80		
		spathulenol	5.10		
		germacrene-B	3.30		
*Phlomis antiatlantica* J.P.Peltier	Morocco/subtropical	No data available.	—	—	—
*Phlomis armeniaca* Willd.	Turkey to Transcaucasus/ temperate	germacrene-D	35.68	Koprulu Canyon National Park, Turkey	[69]
		β-caryophyllene	18.08		
		caryophyllene oxide	13.35		
		(E)-β-farnesene	7.24		
		hexahydrofarnesyl acetone	6.99		
		germacrene-D	24.10	Bolvadin Afyonkarahisar Turkey	[24]
		hexahydrofarnesyl acetone	13.70		
		spathulenol	6.00		
		β-caryophyllene	4.80		
		linalool	3.60		
		β-farnesene	6.20	Kayseri, Turkey	[67]
		germacrene-D	23.40		
		hexadecanoic acid	4.90		
		β-selinene	2.60		
		bicyclogermacrene	2.30		
		germacrene-D	27.22	Turkey	[70]
		β-caryophyllene	16.63		
		(E)-2-hexenal	12.12		
		vinly amly carbinol	5.45		
		benzaldehyde	3.80		
*Phlomis aucheri* Boiss.	W. Iran/ temperate	caryophyllene oxide	33.50	Fars province, Iran	[64]
		β-caryophyllene	27.00		
		β-selinene	10.20		
		germacrene-D	11.10	Iran	[71]
		bicyclogermacrene	6.30		
		spathulenol	6.01		
		β-caryophyllene	5.58		
		neryl acetate	4.58		
		germacrene B	4.53		
		cyclopentane	77.20	Ardebil, Iran	[72]
		n-octacosane	7.30		
		germacrene-D	1.60		
		n-pentacosane	2.50		
		n-hexacosane	2.30		
		germacrene-D	28.31	Province of Ilam, West of Iran	[73]
		γ-elemene	5.46		
		α-pinene	4.81		
		β-caryophyllene	4.96		
*Phlomis aurea* Decne.	Sinai/ subtropical	germacrene-D	51.56	Saint Catherine, Sinai, Egypt	[74]
		trans-β-farnesene	11.36		
		α-pinene	22.96		
		limonene	6.26		
		B-Z-Ocimene	1.85		
*Phlomis bourgaei* Boiss.	East Aegean Islands to SW. Türkiye/subtropical	β-caryophyllene	37.37	Mugla, Turkey	[23]
		(Z)-β-farnesene	15.88		
		germacrene-D	10.97		
		limonene	8.97		
		α-cubebene	7.43		
		β-caryophyllene	21.98	Turkey	[70]
		α-cubebene	16.04		
		germacrene-D	15.12		
		limonene	5.84		
		Β-cubebene	4.59		
*Phlomis bovei* de Noé	Algeria, Morocco, Tunisia/subtropical	germacrene-D	21.45	Megriss Mountain, Eastern Algeria	[75]
		β-caryophyllene	7.05		
		hexahydrofarnesyl acetone	5.84		
		β-bourbonene	2.96		
		caryophyllene oxide	2.41		
*Phlomis brachyodon* (Boiss.) Zohary ex Rech.f.	Lebanon–Syria, Palestine, Saudi Arabia/desert or dry shrubland	γ-muurolene	15.85	Shobak City, Southern Jordan	[76]
		α-humulene	19.00		
		linalool	6.42		
		germacrene-B	4.93		
		γ-himachalene	8.12		
*Phlomis brevibracteata* Turrill	Cyprus/subtropical	caryophyllene oxide	26.00	Geçitköy, North Cyprus	[77]
		β-caryophyllene	21.90		
		α-pinene	4.50		
		isocaryophyllene oxide	5.30		
		limonene	3.20		
*Phlomis brevidentata* H.W.Li	Tibet/temperate	No data available.	—	—	—
*Phlomis brevilabris* Ehrenb. ex Boiss.	Lebanon–Syria/subtropical	No data available.	—	—	—
*Phlomis bruguieri* Desf.	Iran, Iraq, Lebanon–Syria, Turkey/temperate	germacrene-D (%),	60.50	Fars province, Iran	[78]
		γ-elemene	16.50		
		germacrene B	7.10		
		bicyclogermacrene	4.10		
		germacrene-D	23.60	Mazandaran province, North of Iran	[79]
		4-hydroxy-4-methyl-2-pentanone	15.0		
		α-pinene	6.80		
		β-caryophyllene	6.70		
		α-pinene	5.50	Kurdistan province, Iran	[21]
		γ-muurolene	15.50		
		caryophyllene oxide	16.30		
		α-selinene	7.10		
		cis-calamene	6.70		
		caryophyllene oxide	10.56	Bingol, Yelesen village, Turkey	[80]
		β-caryophyllene	8.28		
		β-pinene	9.63		
		α-cubebene	8.89		
		p-cymen-8-ol	4.70		
*Phlomis brunneogaleata* Hub.-Mor.	Turkey/temperate	No data available.	—	—	—
*Phlomis bucharica* Regel	Tadzhikistan to N. Afghanistan/temperate	thymol	20.41	Uzbekistan	[81]
		camphor	14.46		
		1,8-cineole	13.69		
		borneol	9.82		
		hexahydrofarnesyl acetone	7.54		
*Phlomis cancellata* Bunge	Afghanistan, Iran, Transcaucasus, Turkmenistan/temperate	germacrene-D	25.60	Northern Iran	[82]
		α-pinene	6.40		
		heptane	4.30		
		hexahydrofarnesyl acetone	4.10		
		β-caryophyllene	3.60		
		germacrene-D	26.40	North of Soltan-Abad, Province of Khorasan, Iran	[83]
		β-caryophyllene	17.00		
		caryophyllene oxide	10.40		
		α-humulene	6.30		
		α-thujene	6.00		
		hexadecanoic acid	17.30		[84]
		germacrene-D	14.60		
		eudesmol	8.50		
		octacosane	5.60		
		(E)-caryophyllene	5.40		
*Phlomis capitata* Boiss.	Turkey/temperate	No data available.	—	—	—
*Phlomis carica* Rech.f.	SW Turkey/subtropical	No data available.	—	—	—
*Phlomis cashmeriana* Royle ex Benth.	Afghanistan, Pakistan, Tadzhikistan, West Himalaya/temperate	o-cymene	53.80	Khyber Pakhtunkhwa, Pakistan	[85]
		sabinen	24.00		
		α-citral	5.00		
		β-pinene	4.97		
		1,8-cineole (%).	4.40		
*Phlomis cashmirica* Wells	Kashmir/temperate	No data available.			
*Phlomis chimerae* Boissieu	Turkey/subtropical	β-caryophyllene	31.60	Antalya, Tekirova, Çıralı, Turkey	[86]
		α-pinene	11.00		
		germacrene D	6.10		
		limonene	5.50		
		linalool	4.70		
*Phlomis chorassanica* Bunge	NE Iran/temperate	germacrene-D	51.50	Khorassan Province, Iran	[58]
		β-caryophyllene	25.00		
		camphor	2.50		
		β-bourbonene	1.50		
		hexahydrofarnesyl acetone	2.50		
*Phlomis chrysophylla* Boiss.	Lebanon–Syria, Palestine/subtropical	No data available.	—	—	—
*Phlomis cretica* C.Presl	East Aegean Is., Greece, Crete/subtropical	germacrene-D	20.10	Zaros, Crete, Greece	[22]
		β-caryophyllene	17.30		
		α-pinene	9.40		
		linalool	7.50		
		limonene	7.10		
		germacrene-D	47.90	Chania, Crete, Greece	[87]
		α-pinene	11.20		
		β-himachalene	7.30		
		germacrene B	6.40		
		limonene	6.10		
*Phlomis crinita* Cav.	Algeria, Morocco, Spain, Tunisia/subtropical	trans-caryophyllene	40.80	Monastir, Tunisia	[88]
		germacrene-D	39.10		
		β-cadinene	0.90		
		α-copaene	0.39		
		d-3-Caren	0.10		
*Phlomis cyclodon* Knorring	Tadzhikistan/temperate	No data available.	—	—	—
*Phlomis cypria* Post	Cyprus/ subtropical	germacrene-D	20.80	Beşparmak Mountains, North Cyprus	[77]
		β-caryophyllene	37.40		
		α-pinene	6.90		
		limonene	4.40		
		palmito-γ-lactone	4.50		
*Phlomis dincii* Yıld.	Turkey/temperate	No data available.			
*Phlomis drobovii* Popov	Kirgizstan, Tadzhikistan, Uzbekistan/temperate	No data available.	—	—	—
*Phlomis elliptica* Benth.	W and S Iran/temperate	β-farnesene	28.90	Fars province, Iran	[89]
		trans-caryophyllene	20.30		
		β-selinene	10.00		
		α-gurjunene	6.40		
		α-pinene	11.30		
		hexadecanoic acid	19.10	Fars province, Iran	[64]
		linoleic acid	10.20		
		β-selinene	9.90		
		hexahydrofarnesyl acetone	7.30		
		(E)-b-farnesene	6.20		
*Phlomis elongata* Hand.-Mazz.	Iraq/subtropical	No data available.	—	—	—
*Phlomis floccosa* D.Don	Egypt, Crete (Greece), Libya, Tunisia/subtropical	germacrene-D	19.70	Touza, Governorate of Monastir, Tunisia	[90]
		β-caryophyllene	15.50		
		caryophyllene oxide	8.30		
		hexadecanoic acid	7.90		
		carvacrol	6.10		
*Phlomis fruticetorum* Gontsch.	Tadzhikistan/temperate	No data available.	—	—	—
*Phlomis fruticosa* L.	Subtropical Albania, Cyprus, East Aegean Is., Greece, Italy, Greece (Crete), Sardegna, Sicilia, Transcaucasus, Turkey, Ex-Yugoslavia/subtropical	α-pinene	38.90 A, 56.60 B	Bar, Montenegro	[19]
		1,8-cineole	8.00 A, 10.40 B		
		β-caryophyllene	8.70 A, 2.00 B		
		α-thujone	2.0 0A, 1.40 B		
		limonene	2.10 A, 2.20 B		
		germacrene-D	21.40	Central-east Peloponnesus, Greece	[22]
		α-pinene	12.60		
		linalool	8.00		
		β-caryophyllene	12.60		
		-γ-bisabolene	7.10		
		α-curcumene	38.00	Toscolano Maderno, Brescia, Italy	[39]
		caryophyllene oxide	46.00		
		α-cedrene	28.00		
		α-cedrene	12.80		
		trans-sesquisabinene hydrate	3.90		
*Phlomis ghilanensis* K.Koch	N. Iran/temperate	No data available.	—	—	—
*Phlomis grandiflora* H.S.Thomps.	E. Aegean Islands to SW and S Turkey/subtropical	germacrene-D	45.40	Antalya, Elmalı district, Turkey	[86]
		β-caryophyllene	22.80		
		bicyclogermacrene	4.90		
		α-humulene	2.80		
		limonene	2.70		
		β-eudesmol	61.48	Tahtalı-Antalya, Turkey	[91]
		β-curcumene	5.81		
		E-β-farnesene	2.35		
		α-zingiberene	2.18		
		α-cedrene	1.94		
		α-pinene	26.40	Turkey	[70]
		α-cedrene	28.15		
		α-curcumene	13.92		
		β-humulene	7.52		
		germacrene-D	5.40		
*Phlomis herba-venti* L.	Albania, Algeria, Bulgaria, East European Russia, France, Greece, Iran, Iraq, Italy, Kazakhstan, Krym, Lebanon-Syria, Morocco, North Caucasus, Palestine, Portugal, Romania, Sicilia, South European Russi, Spain, Transcaucasus, Tunisia, Turkey, Turkey-in-Europe, Turkmenistan, Ukraine, Ex-Yugoslavia/subtropical	germacrene-D	33.90	Mazandaran province, Northern Iran	[92]
		hexadecanoic acid	12.90		
		α-pinene	9.40		
		14-hydroxy-α-muurolene	4.20		
		β-bourbonene	4.00		
		germacrene-D	31.10	Mazandaran province, Northern Iran	[93]
		T-muurolol	11.0		
		β-caryophyllene	1.70		
		β-bourbonene	1.50		
		α-pinene	7.10		
		germacrene-D	24.50	Orromieyeh, Province, Iran	[94]
		β-farnesene	13.40		
		bicyclogermacrene	14.10		
		α-pinene	13.50		
		hexadecanoic acid	0.10		
		(E)-2-hexenal	17.60	Turkey	[70]
		vinly amyl carbinol	20.44		
		germacrene-D	9.84		
		n-hexanal	4.86		
		3-methylbutanal	4.41		
		n-hexadecanoic acid	68.10	Ankara, Turkey	[24]
		germacrene D	7.20		
		hexahydrofarnesyl acetone	4.00		
		α-cadinol	2.30		
		spathulenol	2.20		
		germacrene-D	11.70	Shanjan Region, Iran	[95]
		β-bourbonene	7.30		
		α-pinene	7.30		
		terpinolene	9.10		
		hexadecanoic acid	7.40		
*Phlomis hypoleuca* Vved.	Kirgizstan, Tadzhikistan/temperate	No data available.	—	—	—
*Phlomis integrifolia* Hub.-Mor.	Turkey/temperate	germacrene-D	20.00	Turkey	[96]
		(Z)-β-farnesene	13.00		
*Phlomis iranica* Joharchi & Vaezi	Iran/temperate	No data available.	—	—	—
*Phlomis isiliae* Yıld.	Turkey/temperate	No data available.	—	—	—
*Phlomis italica* L.	Baleares, Spain/subtropical	No data available.	—	—	—
*Phlomis kotschyana* Hub.-Mor.	Lebanon-Syria, Turkey/subtropical	No data available.	—	—	—
*Phlomis kurdica* Rech.f.	Iran, Iraq, Lebanon–Syria, Palestine, Turkey/temperate	germacrene-D	55.40	Malatya, Turkey	[97]
		(Z)-β-farnesene	11.20		
		hexadecanoic acid	8.40		
		bicyclogermacrene	3.80		
		δ-cadinene	1.90		
		dodecanoic acid	10.00	Kurdistan Province, Iran	[21]
		germacrene-D	9.90		
		trans-caryophyllene	8.70		
		α-selinen	6.30		
		α-cubebene	5.90		
*Phlomis lanata* Willd	Crete, Greece/subtropical	α-Pinene	25.41	Heraklion, Crete, Greece	[17]
		limonene	15.67		
		trans-caryophyllene	8.86		
		γ-muurolene	4.53		
		isocomen	4.91		
*Phlomis lanceolata* Boiss. & Hohen.	SE. Turkey to W. Iran/temperate	germacrene-D	47.00	Ardebil province, Iran	[78]
		(E)-β-farnesene	10.50		
		α-pinene	8.70		
		germacrene B	8.00		
		bicyclogermacrene	5.90		
*Phlomis leucophracta* P.H.Davis & Hub.-Mor.	S Turkey/subtropical	β-caryophyllene	20.20	Alanya district, Alanya Castle, Turkey	[86]
		α-pinene	19.20		
		limonen	11.00		
		nonanal	8.80		
		germacrene-D	4.50		
		linalool	36.40	Turkey	[98]
		spathulenol	8.40		
		caryophyllene oxid	8.40		
		β-caryophyllene	7.90		
		nonanal	4.80		
		limonene	27.86	Haziran, Turkey	[99]
		β-caryophyllene	26.55		
		α-pinene	11.59		
		nonana	4.33		
		β-myrcene	3.75		
		limonene	14.56	Turkey	[70]
		β-caryophyllene	22.45		
		germacrene-D	8.32		
		(E)-2-hexenal	8.74		
		n-hexanal	3.54		
*Phlomis linearifolia* Zakirov	Tadzhikistan, Uzbekistan/temperate	No data available.	—	—	—
*Phlomis linearis* Boiss. & Balansa	Central Turkey/temperate	β-caryophyllene	24.20	Kayseri, Erciyes Mountain, Turkey	[100]
		germacrene-D	22.30		
		caryophyllene oxide	9.20		
		(Z)-β-farnesene	6.60		
		hexahydrofarnesyl acetone	5.80		
		α-pinene	12.50	Sivas Province, Turkey	[101]
		β-caryophyllene	10.70		
		α-cadinol	10.40		
		germacrene-D	8.80		
		acetophenone	7.50		
*Phlomis longifolia* Boiss. & C.I.Blanche	Cyprus, Lebanon–Syria, Turkey/subtropical	caryophyllene oxide	27.20	Hatay, Belen district, Turkey	[102]
		fenchone	18.63		
		1,8-cineole	6.12		
		camphor	4.82		
		α-cubebene	4.11		
*Phlomis lunariifolia* Sm.	Cyprus, Turkey/subtropical	β-caryophyllene	9.00	Icel, Aydincik–Gulnar, Turkey	[103]
		germacrene-D	7.70		
		β-farnesene	6.50		
		hexadecanoic acid	9.70		
		spathuleno	3.90		
*Phlomis lurestanica* Jamzad	Iran/temperate	hexadecane	8.97	Lorestan province, Iran	[104]
		2-dodecnenal	6.57		
		heptadecane	6.32		
		α-pinene	3.13		
		carvacrol	3.50		
*Phlomis lychnitis* L.	France, Morocco, Portugal, Spain/temperate	No data available.	—	—	—
*Phlomis lycia* D.Don	SW Turkey/subtropical	No data available.	—	—	—
*Phlomis majkopensis* (Novopokr.) Grossh.	N Caucasus/temperate	No data available.	—	—	—
*Phlomis mazandaranica* Jamzad	Iran/temperate	No data available.	—	—	—
*Phlomis mindshelkensis* Lazkov	Kazakhstan/temperate	No data available.	—	—	—
*Phlomis monocephala* P.H.Davis	S Turkey/subtropical	germacrene-D	18.92	Mersin Silifke Bahçederesi Village, Turkey	[105]
		(E)-β-farnesene	17.69		
		α-pinene	15.59		
		3-octen-2-one	6.97		
		δ-cadinene	4.27		
		germacrene-D	6.00	Icel, Adicike Gulnar Turkey	[67]
		α-pinene	4.90		
		limonene	3.90		
		β-farnesene	3.10		
		spathulenol	3.00		
*Phlomis nana* C.Y.Wu	Tibet/temperate	No data available.	—	—	—
*Phlomis nissolii* L.	Turkey/subtropical	germacrene-D	33.90	Konya, Turkey	[106]
		bicyclogermacrene	15.30		
		(Z)-β-farnesene	10.70		
		carvacrol	4.20		
		spathulenol	4.00		
		germacrene-D	15.10	Konya, Turkey	[24]
		β-caryophyllene	12.70		
		linalool	11.30		
		hexahydrofarnesyl acetone	11.90		
		spathulenol	4.00		
		germacrene-D	20.73	Turkey	[107]
		(E)-2-hexenal	10.57		
		α-pinene	6.93		
		β-caryophyllene	12.15		
		(E)-β-farnesene	4.05		
*Phlomis nubilans* Zakirov	Uzbekistan/temperate	No data available.	—	—	—
*Phlomis nyalamensis* H.W.Li	Tibet/temperate	No data available.	—	—	—
*Phlomis olgae* Regel	Tadzhikistan, Uzbekistan/ temperate	No data available.	—	—	—
*Phlomis olivieri* Benth.	Iraq to Iran/subtropical	germacrene-D	5.30 to 36.90	Hamedan province, Iran	[108]
		(E)-β-caryophyllene	6.30 to 61.90		
		(E)-β-farnesene	5.10 to 18.40		
		bicyclogermacrene	0.50 to 7.50		
		caryophyllene oxide	1.70 to 10.10		
		germacrene-D	28.10	Tehran Province, Iran	[109]
		β-caryophyllene	16.10		
		α-pinene	11.70		
		β-selinene	10.20		
		bicyclogermacrene	7.40		
		germacrene-D	26.40	Lorestan, Iran	[110]
		bicyclogermacrene	12.70		
		α-pinene	7.70		
		limonene	3.67		
		β-bourbonene	4.00		
		germacrene-D	48.00	Mazandaran province, Northern Iran	[111]
		α-pinene	10.40		
		bicyclogermacrene	4.40		
		germacrene-B	3.80		
		δ-cadinene	2.50		
		germacrene-D	37.60	Yasooj (province of Kohkiloye-Boyerahmad), Iran	[112]
		β-caryophellene	10.00		
		bicyclogermacrene	8.00		
		β-selinene	6.80		
		spathulenol	4.80		
		β-caryophyllene	25.70	Gandoman region, Iran	[113]
		germacrene-D	19.50		
		α-pinene	9.00		
		β-farnesene	9.40		
		caryophyllene oxide	4.20		
		germacrene-D	26.54 to 56.41	Zagros region, Iran	[20]
		bicyclogermacrene	6.38 to 30.55		
		β-caryophyllene	5.32 to 24.52		
		α-pinene	1.29 to 15.53		
		1,8-cineole	1.11 to 4.92		
		germacrene-D	26.43	Azeran and Kamoo in Kashan, Iran	[114]
		β-caryophyllene	20.72		
		elixene	6.58		
		β-trans-farnesene	6.17		
		β-cyclogermacrane	5.04		
*Phlomis oppositiflora* Boiss. & Hausskn.	Turkey/temperate	No data available.	—	—	—
*Phlomis orientalis* Mill.	Iran, Transcaucasus/temperate	No data available.	—	—	—
*Phlomis pachyphylla* Rech.f.	W and SW Iran/temperate	No data available.	—	—	—
*Phlomis persica* Boiss.	Iran/temperate	germacrene-D	38.20	Taleghan Iran	[110]
		bicyclogermacrene	16.30		
		α-pinene	13.30		
		germacrene B	8.80		
		γ-elemene	2.60		
		germacrene-D	26.50	Yasooj (province of Kohkiloye-Boyerahmad), Iran	[112]
		bicyclogermacrene	18.70		
		spathulenol	6.80		
		hexahydrofarnesyl acetone	9.00		
		linoleic acid	6.50		
		germacrene-D	17.20	Gandoman region, Iran	[113]
		γ-elemene	15.40		
		(e)-β-farnesene	9.40		
		α-pinene	9.00		
		caryophyllene oxide	4.20		
*Phlomis physocalyx* Hub.-Mor	Central Turkey/temperate	No data available.	—	—	—
*Phlomis pichleri* Vierh.	Creta, Greece/subtropical	No data available.	—	—	—
*Phlomis platystegia* Post	Israel to Jordan/subtropical	linalool	7.72	Ajloun City, North of Jordan	[76]
		neo-dihydro carveol	3.20		
		viridiflorol	17.39		
		β-eudesmol	13.07		
		ethyl-pentanoate	3.35		
*Phlomis polioxantha* Rech.f.	N Iraq, W and SW Iran/temperate	No data available.	—	—	—
*Phlomis purpurea* L.	Algeria, Morocco, Portugal, Spain/subtropical	No data available.	—	—	—
*Phlomis regelii* Popov	Uzbekistan/temperate	camphene	17.10	Uzbekistan	[115]
		1,8-cineole	15.90		
		β-cymene	7.90		
		limonene	7.40		
		trans-2-hexenal	4.60		
*Phlomis rigida* Labill.	Iran, Iraq, Lebanon–Syria, Turkey/ subtropical	β-caryophyllene	31.20	Turkey	[116]
		β-selinene	13.10		
		α-selinene	2.60		
		α-humulene	3.00		
		hexahydrofarnesyl acetone	1.40		
		(E)-2-hexenal	9.21	Konya Turkey	[105]
		β-caryophyllene	60.23		
		germacrene-D	9.76		
		α-humulene	1.25		
		vinyl amyl carbinol	1.90		
		(Z)-β-ocimene	22.50	Hamadan Province, Iran	[59]
		isobornyl acetate	18.10		
		trans-verbenol	9.30		
		α-pinene	9.20		
		camphene	3.70		
*Phlomis russeliana* (Sims) Lag. ex Benth.	N Turkey/subtropical	β-caryophyllene	23.00	Bolu: Abant lake, Turkey	[103]
		germacrene-D	15.00		
		caryophyllene oxide	8.10		
		hexahydrofarnesyl acetone	3.90		
		α-humulene	1.50		
		α-humulene	26.89	Mazandaran province, Iran	[117]
		mono(2-ethylhexyl) phthalate	20.42		
		caryophyllene	18.74		
		borneol	5.96		
		camphor	4.45		
*Phlomis salicifolia* Regel	Kirgizstan, Tadzhikistan, Uzbekistan/temperate	methyl palmitate	51.15	Uzbekistan	[81]
		hexahydrofarnesyl acetone	7.69		
		methyl linolenate	5.99		
*Phlomis samia* L.	Balkan Peninsula to SW and S Turkey/subtropical	(E)-β-farnesene	20.70	Mount Parnon-Peloponnesus, Greece	[22]
		germacrene-D	6.30		
		βcaryophyllene	5.80		
		spathulenol	3.70		
		caryophyllene oxide	3.20		
		germacrene-D	33.80	Turkey	[116]
		β-caryophyllene	6.40		
		α-copaene	3.20		
		hexahydrofarnesyl acetone	2.80		
		bicyclogermacrene	2.50		
		α-copaene	10.59	Isparta, Turkey	[70]
		β-caryophyllene	15.20		
		germacrene-D	23.44		
		β-bourbonene	7.47		
		δ-cadinene	5.29		
*Phlomis sewerzowii* Regel	Kirgizstan, Uzbekistan/temperate	thymol	35.76	Uzbekistan	[81]
		carvacrol	8.90		
		β-caryophyllene	8.43		
		caryophyllene oxide	8.32		
		hexahydrofarnesyl acetone	8.25		
*Phlomis sieheana* Rech.f.	Central Turkey/temperate	germacrene-D	16.60	Kayseri, Turkey	[67]
		β-farnesene	11.70		
		spathulenol	3.00		
		β-bourbonene	1.50		
		hexahydrofarnesyl acetone	1.90		
		β-caryophyllene	10.80	Hazersah village (Solhan-Bingöl), Turkey	[118]
		germacrene-D	15.60		
		α-pinene	7.80		
		β-farnesene	5.90		
		caryophyllene oxide	2.30		
*Phlomis sintenisii* Rech.f.	E Turkey/temperate	spathulenol	7.00		[96]
*Phlomis spinidens* Nevski	Tadzhikistan/temperate	No data available.	—	—	—
*Phlomis stewartii* Hook.f.	Afghanistan, Pakistan/temperate	No data available.	—	—	—
*Phlomis syriaca* Boiss.	S Turkey to SW Syria/subtropical	No data available.	—	—	—
*Phlomis tathamiorum* R.M.Haber & Semaan	Lebanon–Syria/subtropical	No data available.	—	—	—
*Phlomis tenorei* Soldano	S Italy/subtropical	β-caryophyllene	15.60	Monopoli, Bari Province, Italy	[119]
		hexadecanoic acid	12.80		
		germacrene-D	8.90		
		caryophyllene oxide	6.70		
		α-thujone	5.50		
*Phlomis tenuis* Knorring	Kazakhstan/temperate	No data available.	—	—	—
*Phlomis thapsoides* Bunge	Tadzhikistan, Uzbekistan/temperate	phenylethyl alcohol	6.81	Surkhondaryo region, Uzbekistan	[120]
		trans-3-hexenol	5.55		
		1-octen-3-ol	5.10		
		α-cadinol	4.92		
		α-muurolol	4.67		
*Phlomis tomentosa* Regel	Tadzhikistan/temperate	No data available.	—	—	—
*Phlomis trineura* Rech.f.	N Afghanistan/temperate	No data available.	—	—	—
*Phlomis viscosa* Poir.	Lebanon–Syria, Palestine, Turkey/subtropical	germacrene-D	4.70	Bașkonuș Mountains, Turkey	[121]
		β-caryophyllene	24.40		
		α-monocyclo farnesyl acetone	16.44		
		α-humulene	6.10		
		alloaromadendrene	11.00		
		germacrene-B	20.15	Ajloun City, North of Jordan	[76]
		e-caryophyllene	9.57		
		α-humulene	2.92		
		γ-muurolene	7.37		
		himachalene epoxide	4.69		
*Phlomis zenaidae* Knorring	Uzbekistan/temperate	No data available.	—	—	—

## Data Availability

Not applicable.
